# Hepatotoxicity of ICI monotherapy or combination therapy in HCC: A systematic review and meta-analysis

**DOI:** 10.1371/journal.pone.0323023

**Published:** 2025-05-29

**Authors:** Yuping Lu, Jing Lin, Yufeng Lu, Luping Lin, Shicheng Zheng, Yu Chen, Sha Huang

**Affiliations:** 1 Department of Medical Oncology, Clinical Oncology School of Fujian Medical University, Fujian Cancer Hospital, Fujian, PR China; 2 School of Mathematics and Computer Science, Fuzhou University, Fujian, PR China; 3 School of Basic Medical Sciences, Fujian Medical University, Fujian, PR China; University of Split Faculty of Medicine: Sveuciliste u Splitu Medicinski fakultet, CROATIA

## Abstract

**Background:**

The aim of this study was to reveal the hepatotoxicity profile of different immune checkpoint inhibitor (ICI) used strategies in patients with Hepatocellular carcinoma (HCC) by meta-analysis.

**Methods:**

Literature was searched through PubMed, Cochrane, Embase, and Web of Science up to October 14, 2023, and the subject terms were “Carcinoma, Hepatocellular” and “Immune Checkpoint Inhibitors”. The main observations were alanine aminotransferase (ALT) and aspartate aminotransferase (AST). ALT and AST were graded according to CTCAE.

**Results:**

A total of 32 studies with 7662 patients were included in the analysis. The results of meta-analysis showed that among different ICI treatment regimens, ICI monotherapy had the lowest incidence of any grade of ALT and AST elevation, and the highest for ICI+multikinase inhibitor (MKI); ICI+anti-VEGFR/VEGFA and ICI monotherapy had a lower incidence of grade ≥3 ALT and AST elevations, while ICI + MKI, dual immunotherapy, and dual immunotherapy+MKI had a higher incidence of grade ≥3 ALT and AST elevations; ICI monotherapy was more prone to any grade ALT elevation than placebo, and ICI monotherapy was more prone to ≥ 3 grade AST elevation than MKI; combination immunotherapy was more prone than MKI to any grade ALT and AST elevations; in grade ≥3 ALT and AST elevations, combination immunotherapy was similar to ICI monotherapy and MKI; ICI + MKI was more likely to have grade ≥3 ALT.

**Conclusion:**

ICI monotherapy was more likely to cause severe hepatotoxicity than MKI. Combination immunotherapy treatment increased the incidence of hepatotoxicity compared to monotherapy, and ICI + MKI was prone to develop severe hepatotoxicity.

## Introduction

Hepatocellular carcinoma (HCC) is currently the sixth most common malignant tumor, and it is mostly detected at an advanced stage [1]. Since HCC is not sensitive to conventional chemotherapy [2], the treatment of hepatocellular carcinoma has always been a major challenge. In 2008, a phase 3 trial named SHARP first verified the efficacy of sorafenib in HCC, and since then, HCC has entered the era of targeted therapy [3]. However, no new drugs for hepatocellular carcinoma treatment were introduced for nearly 10 years, and the treatment of HCC entered into stagnation again. In recent years, with the progress of drug research and development technology, various antitumour drugs have been introduced, which greatly changed the current status of antitumour therapy. The birth of immune checkpoint inhibitors (ICIs) is a landmark event in the treatment of HCC. ICIs are designed to reactivate T-cell-mediated antitumour immunity by blocking the immune checkpoint pathway, reversing the phenomenon of immune escape and thus promoting tumor cell death.

In 2019, the IMBRAVE 150 study established for the first time that atilizumab in combination with bevacizumab was superior to sorafenib in the treatment of HCC, leading to FDA approval as a first-line treatment option for HCC [4]. The publication of CheckMate040 results confirmed the therapeutic efficacy of nivolumab in combination with ipilimumab in the treatment of HCC and was approved by the FDA as a first-line regimen for HCC treatment [5]. Currently, ICI monotherapy, ICI+multikinase inhibitor (MKI), ICI+anti-VEGFR/VEGFA, and dual immunotherapy in HCC are hot topics. However, many problems have arisen. Immune activation caused by ICIs causes many adverse effects, And liver injury is a common adverse effect [6]. MKI, anti-VEGFR/VEGFA antibodies, and anti-CTLA4 antibodies also cause liver injury [7,8], so ICI-based combination therapy seems to increase the occurrence of liver injury [9]. Meanwhile, HCC patients are also prone to elevated liver enzymes due to their own liver tumors or combined HBV infection [10]. In a meta-analysis of 117 trials, the incidence of elevated liver enzymes in HCC compared to other solid tumors was increased twofold[11], which suggests that in addition to focusing on therapeutic efficacy, the incidence of drug-induced liver injury should also be focused on in the treatment of HCC and that drugs susceptible to liver injury should be avoided.

Currently, there is a lack of reports on the direct comparison of the occurrence of liver injury due to ICI monotherapy and various ICI-based combination therapies in HCC. Therefore, we aimed to reveal the differences in liver injury due to different ICI regimens in HCC treatment by meta-analysis.

## Methods

This study protocol has been registered in the international prospective registry of the systematic review PROSPERO (ID: CRD42023457815).

### Search strategy

We searched the PubMed, Embase, Cochrane Library, and Web of Science databases with an October 14, 2023 deadline for literature publication. A combination of subject terms and free terms was used for the literature search. Subject terms were Carcinoma, Hepatocellular and Immune Checkpoint Inhibitors, and the specific search protocol is described in S1 File. We also manually retrieved references for studies selected by electronic search to identify other relevant studies.

### Inclusion and exclusion criteria

The inclusion criteria were as follows: 1) pathologically confirmed hepatocellular carcinoma, excluding intrahepatic cholangiocarcinoma and combined hepatocellular-cholangiocarcinoma; 2) “ ≥ 18 years old; 3) prospective study; 4) treatment regimens containing ICIs in at least one group; 5) English-language literature.

Exclusion criteria: 1) studies in the dose escalation phase; 2) number of cases less than 10; 3) nonclinical studies, reviews, letters, pathologic studies, reviews, meta-analyses, case reports, case series, etc.; 4) literature in other languages; 5) no relevant data; 6) no relevant populations; 7) original literature unavailable.

### Literature screening and data extraction

Two investigators independently screened the literature for final inclusion of studies based on inclusion and exclusion criteria and then independently extracted information. A standardized spreadsheet for information extraction was created prior to information extraction, and data extracted included basic information [article title, first author, year of publication, national clinical trial (NCT) registry number, type of study, method of intervention, sample size] and primary outcome indicators [alanine aminotransferase (ALT) and aspartate aminotransferase (AST)]. The degree of ALT and AST elevation was categorized according to the CTCAE 5.0 classification as 1) Grade 1: > 1–3 upper limit of normal (ULN); 2) Grade 2: > 3–5 ULN; 3) Grade 3: > 5–20 ULN; 4) >20 ULN; and 5) death. Of these, grade ≥3 elevated ALT or AST was defined as severe liver injury.

### Quality assessment

The quality of evidence was scored using the MINORS scoring system for non-randomized controlled studies and the Cochrane Risk of Bias Assessment Tool for randomized controlled clinical studies [12]. Funnel plots were used to assess literature bias.

### Statistical analysis

Single-arm meta-analysis was performed using the “metaprop” package of RStudio for ALT and AST elevations in all included literature with ICI or ICI combination therapy, and RevMan software for ALT and AST elevations in RCT studies. For I^2 ^≥ 50%, heterogeneity was considered significant, and a random effects model was used to pool effect sizes. I^2^ was used to quantify heterogeneity in meta-analysis. When I^2^ < 50%, fixed effects models were used to pool effect sizes. Odds ratio (OR) with 95% confidence interval (CI) was calculated as the measure of effect size.

## Results

### Literature screening process and literature characterization

**Fig 1:**
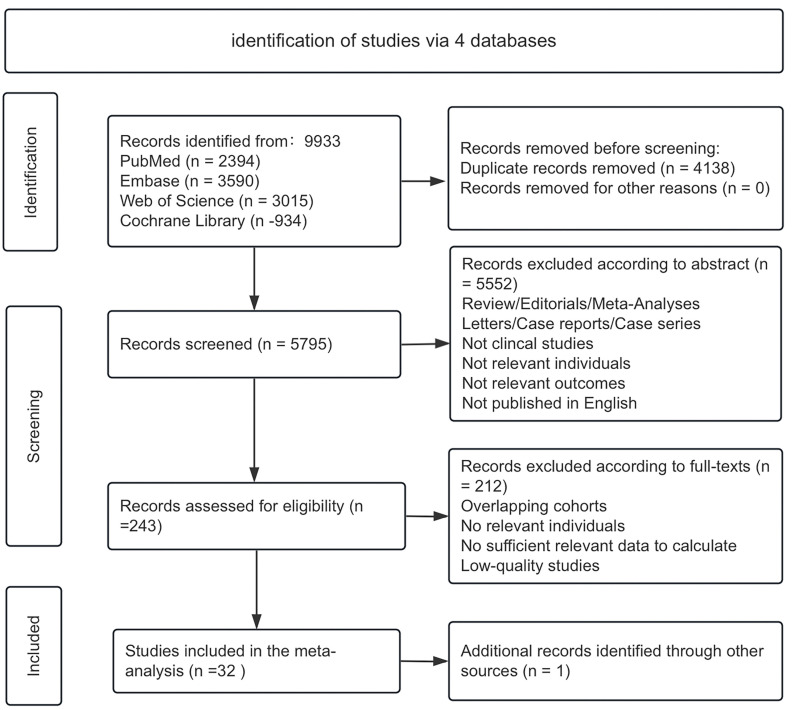
Study inclusion plot.

We obtained a total of 9,932articles by searching the PubMed, Embase, Web of Science, and Cochrane Library databases. Additionally, we additionally obtained 1 articles by reading the article’s reference. The screening process is shown in Fig 1. Ultimately, 32 articles were included in the analysis [13–44], totaling 7662 patients. Of these, 12 were RCTs, and 20 were single-arm clinical studies. The characteristics of the included studies are shown in Table 1 and S1 Fig. Of these, CheckMate 040 had four different subgroups, and because the data were not duplicated, they were considered four different studies included in the analysis in this study [5,25,27,45].

**Table1:** Characteristics of included studies.

First author	Year	NCT	Number of patients	Group1	Group2、3	Study type	MINORS Score
Ghassan K.Abou-Alfa	2022	NCT03298451	1150	Dual immunotherapy	ICI monotherapy, MKI	RCT	
philippe merle	2023	NCT02702401	413	ICI monotherapy	Placebo	RCT	
Ann Lii Cheng	2022	NCT03434379	485	ICI+anti-VEGFR/VEGFA	MKI	RCT	
Ahmed Omar Kaseb	2022	NCT03222076	27	Dual immunotherapy	ICI monotherapy	RCT	
Robin Kate Kelley	2022	NCT03755791	824	ICI + MKI	MKI	RCT	
Michael S Lee	2020	NCT02715531	222	ICI+anti-VEGFR/VEGFA	ICI monotherapy	RCT	
Thomas Yau	2022	NCT02576509	730	ICI monotherapy	MKI	RCT	
Zhenggang Ren	2021	NCT0394440	565	ICI+anti-VEGFR/VEGFA	MKI	RCT	
Shukui Qin	2023	NCT03062358	452	ICI monotherapy	Placebo	RCT	
Shukui Qin	2023	NCT03764293	491	ICI + MKI	MKI	RCT	
Shukui Qin	2023	NCT03412773	662	ICI monotherapy	MKI	RCT	
Thomas Yau	2023	NCT01658878	71	ICI + MKI	Dual immunotherapy+MKI	RCT	
Anthony B El-Khoueiry	2017	NCT01658878	214	ICI monotherapy		Single arm	12
Thomas Yau	2020	NCT01658878	49	Dual immunotherapy		Single arm	12
Masatoshi Kudo	2021	NCT01658878	49	ICI monotherapy		Single arm	12
Richard S, Finn	2020	NCT03006926	100	ICI + MKI		Single arm	12
Jianming Xu	2021	–	190	ICI + MKI		Single arm	12
Masatoshi Kudo	2022	NCT02702414	104	ICI monotherapy		Single arm	12
Lynn G.Feun	2019	NCT02658019	29	ICI monotherapy		Single arm	12
Yung-Jue Bang	2020	NCT02572687	28	ICI+anti-VEGFR/VEGFA		Single arm	12
Zhenggang Ren	2021	NCT02989922	217	ICI monotherapy		Single arm	12
Chun Han	2022	NCT04172571	31	ICI + MKI		Single arm	12
Xiaofeng Chen	2022	NCT04052152	20	ICI + MKI		Single arm	12
Zhengang Ren	2023	NCT03419897	249	ICI monotherapy		Single arm	12
A.R.He	2018	NCT02383212	26	ICI monotherapy		Single arm	12
Haifeng Lin	2022	ChiCTR1900028295	31	ICI + MKI		Single arm	12
Zev A.Wainberg	2017	NCT01693562	40	ICI monotherapy		Single arm	12
Lijun Wang	2023	NCT04042805	36	ICI + MKI		Single arm	12
Shi-xue Laoguo	2023	–	25	ICI + MKI		Single arm	12
Baocai Xing	2022	NCT04542837	55	Dual immunotherapy+MKI		Single arm	12
Wen Zhang	2022	NCT03825705	19	ICI + MKI		Single arm	12
Yujing Xin	2023	–	58	ICI + MKI		Single arm	12

### Results of single-arm meta-analysis on ICI monotherapy or combination therapy

**Fig 2:**
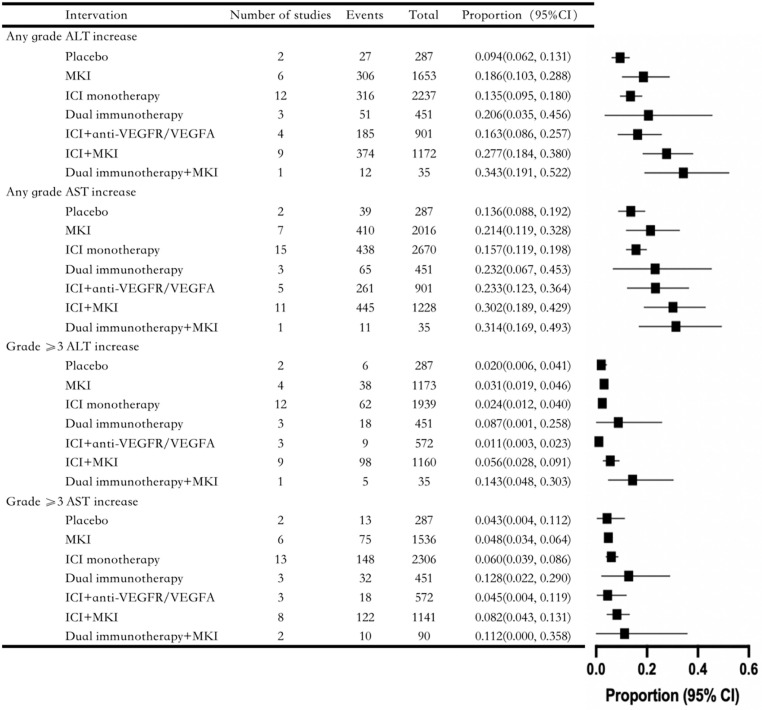
Results of single-arm meta-analysis on ICI monotherapy or combination therapy.

As shown in Fig 2., in the occurrence of liver injury in all grades, dual immunotherapy+MKI and ICI + MKI were most likely to have elevated ALT and AST; MKI, ICI monotherapy, and dual immunotherapy were lower, and all of these regimens had a higher incidence of liver injury than placebo (best supportive care, BSC). In grade ≥3 liver injury, dual immunotherapy + MKI, dual immunotherapy, and ICI + MKI were prone to grade ≥3 ALT and AST elevations; The incidence of grade ≥3 ALT and AST elevations was lower with ICI monotherapy, MKI, and ICI+anti-VEGFR/VEGFA. Interestingly, ICI+anti-VEGFR/VEGFA grade ≥3 had a lower incidence of ALT and AST elevations than placebo.

### Further meta-analysis of RCT studies on liver injury with different ICI treatment regimens

1 **Incidence of Liver Injury at Any Grade of ICI Monotherapy vs. MKI/placebo**

**Fig 3:**
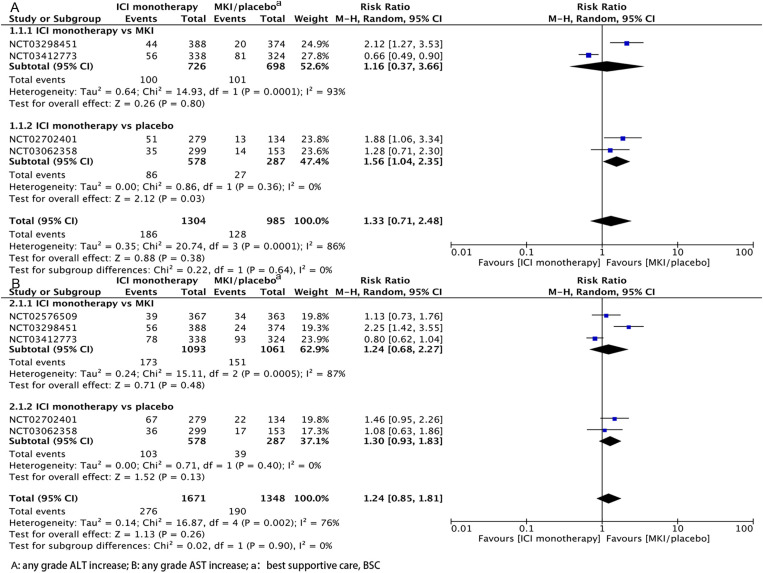
Hepatotoxicity of ICI monotherapy vs MKI/placebo at any grade.

In order to directly compare the occurrence of liver injury among different ICI treatment regimens, we performed further analysis on 12 of these RCT articles. As shown in Fig 3A., the rate of any grade ALT elevation was similar for ICI monotherapy compared to MKI/placebo (RR, 1.33; 95% CI, 0.71–2.48), and further subgroup analyses showed that ICI monotherapy was more likely to have any grade ALT elevation than placebo (RR, 1.56; 95% CI, 1.04–2.35), whereas ICI monotherapy and MKI had similar rates of any grade ALT elevation without an increased incidence of liver injury (RR, 1.16; 95% CI, 0.37–3.66). As shown in Fig 3B, the rate of any grade AST elevation was similar with ICI monotherapy compared with MKI/placebo (RR, 1.24; 95% CI, 0.85–1.81), and further subgroup analyses showed that the rate of any grade AST elevation with ICI monotherapy was similar to that of both MKI (RR, 1.24; 95% CI, 0.68–2.27) and placebo (RR, 1.30; 95% CI, 0.93–1.83), and the difference was not statistically significant. The funnel plot is in S2 Fig.

2 **Incidence of Grade ≥3 Liver Injury with ICI Monotherapy vs. MKI/placebo**

**Fig 4:**
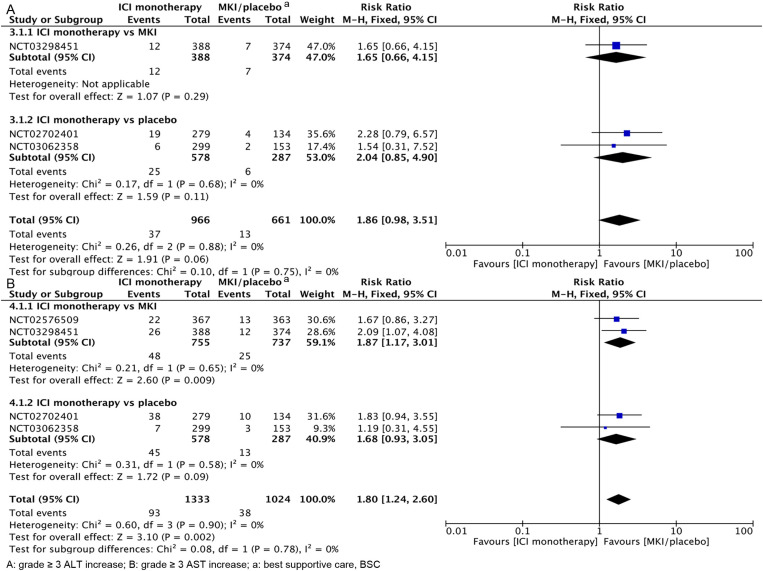
Hepatotoxicity of ICI monotherapy vs MKI/placebo at grade ≥3.

As shown in Fig 4A, ICI monotherapy had a similar rate of grade ≥3 ALT elevation to MKI/placebo (RR, 1.86; 95% CI, 0.98–3.51). Further subgroup analysis showed that ICI monotherapy had a similar rate of grade ≥3 ALT elevation compared with MKI (RR, 1.65; 95% CI, 0.66–4.15) and placebo (RR, 2.04; 95% CI, 0.85–4.90). As shown in Fig 4B, ICI monotherapy was more likely to have grade ≥3 AST elevations than MKI/placebo (RR, 1.80; 95% CI, 1.24–2.60), and further subgroup analyses showed that ICI monotherapy was more likely to have grade ≥3 AST elevations than MKI (RR, 1.87; 95% CI. 1.17–3.01), but the rate of grade ≥3 AST elevation was similar between ICI monotherapy and placebo (RR, 1.68; 95% CI, 0.93–3.05). The funnel plot is in S3 Fig.

3 **Incidence of Liver Injury at Any Grade of Combination Immunotherapy vs. ICI/MKI Monotherapy**

**Fig 5:**
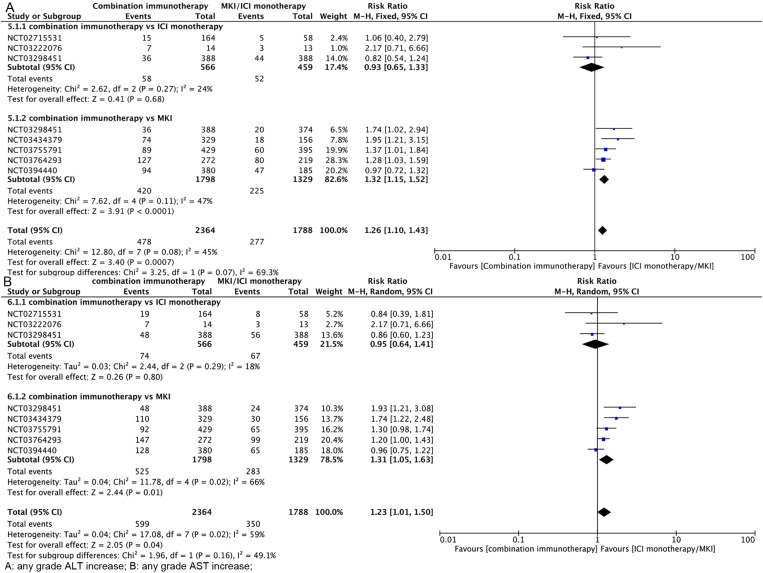
Hepatotoxicity of combination immunotherapy vs ICI monotherapy/MKI at any grade.

As shown in Fig 5A, combination immunotherapy was more likely to have any grade of ALT elevation than ICI/MKI monotherapy (RR, 1.26; 95% CI, 1.10–1.43); further subgroup analyses showed that combination immunotherapy had a similar rate of any grade of ALT elevation as ICI monotherapy (RR, 0.93; 95% CI, 0.65–1.33), but was more likely to have any grade of ALT elevation than MKI (RR, 1.32; 95% CI, 1.15–1.52). As shown in Fig 5B, combination immunotherapy was more likely than ICI/MKI monotherapy to have any grade of AST elevation (RR, 1.23; 95% CI, 1.01–1.50); further subgroup analyses showed that the rate of any grade of AST elevation with combination immunotherapy was similar to that with ICI monotherapy (RR, 0.95; 95% CI, 0.64–1.41), but was more likely than MKI to have any grade of AST elevation (RR, 1.31; 95% CI, 1.05–1.63). The funnel plot is in S4 Fig.

4 **Incidence of Grade ≥3 Liver Injury with Combination Immunotherapy vs. ICI/MKI Monotherapy**

**Fig 6:**
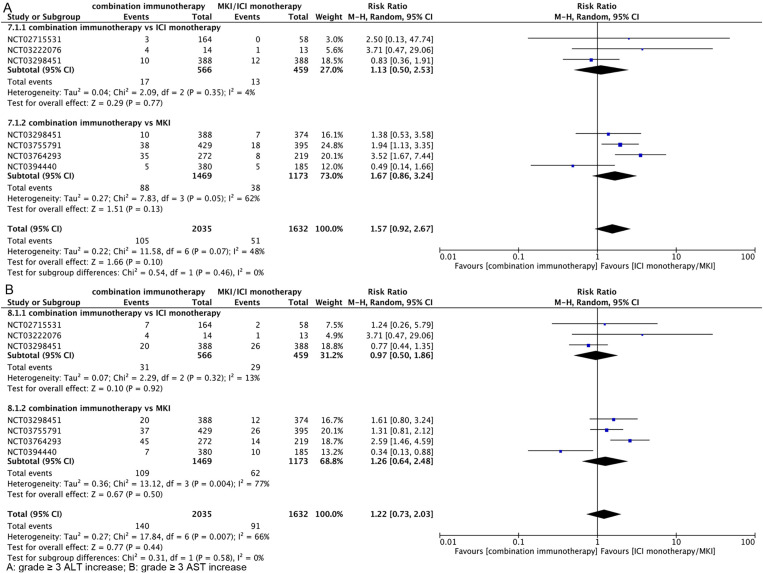
Hepatotoxicity of combination immunotherapy vs ICI monotherapy/MKI at grade ≥3.

As shown in Fig 6A, the incidence of grade ≥3 ALT elevation was similar between combination immunotherapy and ICI/MKI monotherapy (RR, 1.57; 95% CI, 0.92–2.67), and further subgroup analyses showed that the grade ≥3 ALT elevation rate was similar to that of ICI monotherapy (RR, 1.13; 95% CI, 0.50–2.53) and MKI (RR, 1.67; 95% CI, 0.86–3.24) treatments and did not increase the incidence of hepatic injury. As shown in Fig 6B, the incidence of grade ≥3 AST elevation was similar between combination immunotherapy and ICI/MKI monotherapy (RR, 1.22; 95% CI, 0.73–2.03), and further subgroup analyses showed that the grade ≥3 AST elevation rate was similar to ICI monotherapy (RR, 0.97; 95% CI, 0.50–1.86) and MKI (RR, 1.26; 95% CI, 0.64–2.48). The funnel plot is in S5 Fig.

5 **Incidence of grade ≥3 liver injury with different ICI combination regimens vs. MKI/ICI monotherapy**

**Fig 7:**
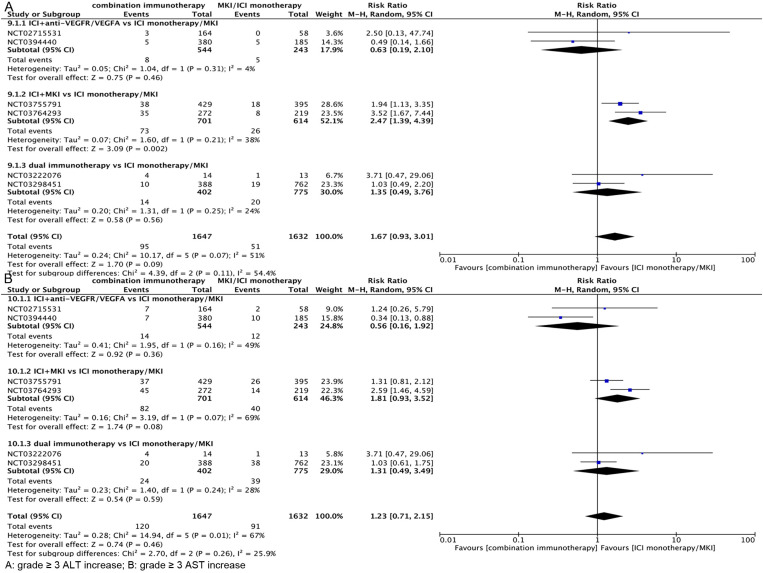
Grade ≥3 Hepatotoxicity in different combination immunotherapy regimens vs ICI monotherapy/MKI.

Because there was no difference in the rate of ALT and AST elevation between combination immunotherapy either with MKI or ICI monotherapy, to further analyse the difference in the occurrence of grade ≥3 liver injury between combination immunotherapy and ICI/MKI monotherapy, we analysed the different immune combination regimens with ICI/MKI monotherapy treatment for differences in liver injury. As shown in Fig 7A, in further subgroup analyses, grade ≥3 ALT elevation was more likely to occur with ICI + MKI than with ICI/MKI monotherapy (RR, 2.47; 95% CI, 1.39–4.39), whereas ICI+anti-VEGFR/VEGFA (RR, 0.63; 95% CI, 0.19–2.10) and dual immunotherapy (RR, 1.35; 95% CI, 0.49–3.76) had a similar incidence of grade ≥3 ALT elevation compared with ICI/MKI monotherapy. As shown in Fig 7B, in further subgroup analyses, the incidence of grade ≥3 AST elevations was similar for ICI + MKI (RR, 1.81; 95% CI, 0.93–3.52), ICI+anti-VEGFR/VEGFA (RR, 0.56; 95% CI, 0.16–1.92), and dual immunotherapy (RR, 1.31; 95% CI. 0.49–3.49) had a similar incidence of grade ≥3 AST elevation compared with ICI/MKI monotherapy. The funnel plot is in S4 Fig.

## Discussion

Currently, the main ICIs used clinically for the treatment of HCC are anti-PD-1/PD-L1 antibodies and anti-CTLA antibodies [46]. Clinically, both anti-PD-1/PD-L1 antibodies and anti-CTLA antibodies have been reported in terms of hepatotoxicity, which is mainly characterized by elevated ALT, AST and bilirubin [47]. ICI-induced liver injury is mediated primarily by immune mechanisms and most often presents as hepatocellular injury, a type of injury that is not characteristic on imaging but is primarily characterized by elevated serum ALT and/or AST [48]. ICI monotherapy mainly refers to anti-PD-1/PD-L1 antibodies, including but not limited to nivolumab, pembrolizumab, ipilimumab, cemiplimab, and atezumab [5], and the hepatotoxicity of PD-1 inhibitors has been reported in the literature to be between 1% and 3% [49]. In our meta-analysis, the incidence of liver injury with ICI monotherapy was higher than that reported in the literature, with 13.5% and 15.7% of any grade ALT and AST elevations, respectively, and 2.4% and 6.0% of ≥ grade 3 ALT and AST elevations, respectively. Liver injury was also compared between ICI monotherapy and placebo. ICI monotherapy was more likely to have any grade of ALT elevation than placebo but did not increase the incidence of any grade of AST and ≥ grade 3 ALT and AST. This demonstrates that although ICI monotherapy has an increased risk of liver injury in the treatment of HCC, it does not increase the probability of severe liver injury in patients with HCC and is generally safe.

MKI monotherapies, such as sorafenib, lenvatinib, regorafenib and cabozantinib, have been approved for the treatment of HCC [50]. A large meta-analysis of MKI-induced hepatotoxicity showed that the incidence of MKI-induced hepatic injury was 23–40% in patients treated with MKIs [51], which is a relatively common adverse event. This also appears to be higher than the hepatotoxicity reported with ICIs. In our meta-analysis, the incidence of any grade ALT and AST elevation was 18.6% and 21.4%, respectively, and the incidence of grade ≥3 ALT and AST elevation was 3.1% and 4.8%, respectively, for MKIs. Our pooled incidence of hepatic injury for MKIs is comparable to what has been reported in the literature, which also seems to have a higher rate of hepatic injury than ICIs monotherapy. However, our meta-analysis of 12 of these RCT articles yielded very interesting results in that any grade ALT, AST, and grade ≥3 ALT elevations were similar for MKI as for ICI monotherapy, and grade ≥3 AST elevations were even more likely to be seen for ICI monotherapy than for MKI, which is contrary to our existing knowledge that ICI monotherapy has a lower incidence of liver injury than MKI. It should be noted that this article is currently the first meta-analysis of liver injury in HCC treatment with ICIs versus MKIs, and the conclusions drawn herein are subject to validation in subsequent large prospective studies of liver injury in HCC treatment with ICIs versus MKIs.

Although the overall safety of ICI monotherapy in the treatment of HCC was confirmed in our study, the survival benefit and efficacy of ICI monotherapy in HCC were low [52]. Resistance to ICIs can be attributed to factors such as immune tolerance in the liver as well as tumor-induced immune escape, and thus, there is an urgent need to explore therapeutic modalities that can ameliorate this phenomenon. Combination immunotherapy has become a hot topic. Anti-CTLA antibodies and anti-PD-1/PD-L1 antibodies have synergistic effects on each other, and the combination of the two can enhance each other’s antitumour effects [53]. In tumor patients, the production of VEGF-A in the tumor microenvironment enhances the expression of PD-1 and other inhibitory checkpoints associated with CD8 + T-cell exhaustion, which is reversed by anti-VEGF/VEGFR therapy and enhances the antitumour effects of anti-PD-1/PD-L1 antibodies [54]. Meanwhile, MKI has been approved for the treatment of HCC, and ICI combined with MKI treatment can increase its antitumour effect [4]. Based on the above principles, three ICI-based immune combination therapies, dual immunotherapy, ICI+anti-VEGFR/VEGFA, and ICI + MKI, have become research hotspots for HCC treatment. Our meta-analysis revealed that combination immunotherapy did not increase the incidence of any grade and grade ≥3 ALT and AST compared to ICI monotherapy. Combination immunotherapy was more likely to have any degree of ALT and AST elevation compared to MKI, but both had similar rates of ALT and AST elevation at grade ≥3. Thus, we further analysed the differences in liver injury between different combination immunotherapy regimens and monotherapy (ICI monotherapy/MKI) treatment and found that dual immunotherapy, ICI+anti-VEGFR/VEGFA, did not result in an increased incidence of grade ≥3 ALT and AST, whereas ICI + MKI was more likely to result in grade ≥3 ALT elevation compared to monotherapy. It should be noted that the two studies included here were the COSMIC-312[55]and CARES-310[27]studies, and the control group was MKI monotherapy; thus, we can conclude that combination immunotherapy increases the incidence of liver injury compared to MKI treatment, and ICI + MKI is more prone to severe liver injury compared to MKI, which should be noted in clinical practice.

Currently, there are three main modalities regarding the clinical application of combination immunotherapy in HCC: dual immunotherapy, ICI+anti-VEGFR/VEGFA, and ICI + MKI. The HIMALAMA study confirmed the statistically significant OS advantage of dual immunotherapy over MKI monotherapy [56]. The IMBRAVE150[9] and ORIENT-32[57] studies confirmed that ICI+anti-VEGFR/VEGFA had longer PFS and OS than MKI, and ICI+anti-VEGFR/VEGFA significantly prolonged PFS compared to immune monotherapy in HCC patients in the G030140[58] study. Previous meta-analyses have also demonstrated that anti-PD-1/PD-L1 antibodies in combination with anti-VEGFR/VEGFA prolonged survival time and improved treatment efficacy in HCC patients [59,60]. The COSMIC-312 study confirmed that ICI + MKI had a longer PFS and higher DCR than MKI alone [55]. Thus, all three current combination therapies result in a survival benefit for HCC patients, and lower hepatotoxicity becomes very important in the treatment of HCC. In our meta-analysis, dual immunotherapy and ICI+anti-VEGFR/VEGFA did not increase the incidence of hepatotoxicity compared to monotherapy, and because of their better survival benefit, they can be prioritized as a priority for HCC patients in the clinic. On the other hand, ICI + MKI increases the incidence of serious liver injury events compared with MKI monotherapy and thus should be chosen with caution in the treatment of HCC.

In summary, ICI monotherapy was relatively safe and did not increase the incidence of severe liver injury events, and among the immune combination therapies, ICI + MKI was associated with an increased risk of severe liver injury compared with MKI monotherapy, whereas dual immunotherapy and ICI+anti-VEGFR/VEGFA did not increase liver injury. However, the population in this meta-analysis was mostly global, and there were almost no reports on the differences in the occurrence of liver injury in patients with HCC of different gender and age characteristics in the original studies, so this meta-analysis did not have further subgroup analyses on the demographic characteristics, which is one of the shortcomings of this paper. At the same time, some of the studies may be underrepresented due to their small sample size. In the future, it is hoped that future clinical studies will make up for these deficiencies. Generally speaking, This article is a meta-analysis, and the results of this article need to be validated in subsequent prospective clinical studies.

## Supporting information

S1 File Draft of search strategy (DOCX)

S2 Fig Funnel plot of any grade liver injury for ICI Monotherapy vs. MKI/placebo. (TIF)

S3 Fig Funnel plot of any grade liver injury for ICI Monotherapy vs. MKI/placebo. (TIF)

S4 Fig Funnel plot of grade ≥3 liver injury for ICI Monotherapy vs. MKI/placebo. (TIF)

S5 Fig Funnel plot of any grade liver injury for Combination Immunotherapy vs. ICI/MKI Monotherapy. (TIF)

S6 Fig Funnel plot of grade ≥3 liver injury for Combination Immunotherapy vs. ICI/MKI Monotherapy. (TIF)

S7 Fig Funnel plot of grade ≥3 liver injury for different ICI combination regimens vs. MKI/ICI monotherapy. (TIF)
