# Supplementary material for: Hepatotoxicity of ICI monotherapy or combination therapy in HCC: A systematic review and meta-analysis
Source: PLoS One. 2025 May 29;20(5):e0323023. doi: 10.1371/journal.pone.0323023 (PMC12121757; doi:10.1371/journal.pone.0323023)
Supplement: S1 File — (DOCX) [file pone.0323023.s001.docx]

**S1 File.**

**Draft of search strategy**

**Search strategy in PubMed**

PubMed search:

(("Carcinoma, Hepatocellular"[Mesh]) OR (((((((((((((((((((Carcinoma, Hepatocellular[Title/Abstract]) OR (Carcinomas, Hepatocellular[Title/Abstract])) OR (Hepatocellular Carcinomas[Title/Abstract])) OR (Liver Cell Carcinoma, Adult[Title/Abstract])) OR (Liver Cancer, Adult[Title/Abstract])) OR (Adult Liver Cancer[Title/Abstract])) OR (Adult Liver Cancers[Title/Abstract])) OR (Cancer, Adult Liver[Title/Abstract])) OR (Cancers, Adult Liver[Title/Abstract])) OR (Liver Cancers, Adult[Title/Abstract])) OR (Liver Cell Carcinoma[Title/Abstract])) OR (Carcinoma, Liver Cell[Title/Abstract])) OR (Carcinomas, Liver Cell[Title/Abstract])) OR (Cell Carcinoma, Liver[Title/Abstract])) OR (Cell Carcinomas, Liver[Title/Abstract])) OR (Liver Cell Carcinomas[Title/Abstract])) OR (Hepatocellular Carcinoma[Title/Abstract])) OR (Hepatoma[Title/Abstract])) OR (Hepatomas[Title/Abstract]))) AND (("Immune Checkpoint Inhibitors"[Mesh]) OR (((((((((((((((((((((((((((((((((((((((((((((Immune Checkpoint Inhibitors[Title/Abstract]) OR (Checkpoint Inhibitors, Immune[Title/Abstract])) OR (Immune Checkpoint Inhibitor[Title/Abstract])) OR (Checkpoint Inhibitor, Immune[Title/Abstract])) OR (Immune Checkpoint Blockers[Title/Abstract])) OR (Checkpoint Blockers, Immune[Title/Abstract])) OR (Immune Checkpoint Blockade[Title/Abstract])) OR (Checkpoint Blockade, Immune[Title/Abstract])) OR (Immune Checkpoint Inhibition[Title/Abstract])) OR (Checkpoint Inhibition, Immune[Title/Abstract])) OR (PD-L1 Inhibitors[Title/Abstract])) OR (PD L1 Inhibitors[Title/Abstract])) OR (PD-L1 Inhibitor[Title/Abstract])) OR (PD L1 Inhibitor[Title/Abstract])) OR (Programmed Death-Ligand 1 Inhibitors[Title/Abstract])) OR (Programmed Death Ligand 1 Inhibitors[Title/Abstract])) OR (PD 1 PD L1 Blockade[Title/Abstract])) OR (CTLA-4 Inhibitors[Title/Abstract])) OR (CTLA 4 Inhibitors[Title/Abstract])) OR (CTLA-4 Inhibitor[Title/Abstract])) OR (CTLA 4 Inhibitor[Title/Abstract])) OR (Cytotoxic T-Lymphocyte-Associated Protein 4 Inhibitors[Title/Abstract])) OR (Cytotoxic T Lymphocyte Associated Protein 4 Inhibitors[Title/Abstract])) OR (Cytotoxic T-Lymphocyte-Associated Protein 4 Inhibitor[Title/Abstract])) OR (Cytotoxic T Lymphocyte Associated Protein 4 Inhibitor[Title/Abstract])) OR (PD-1 Inhibitors[Title/Abstract])) OR (PD 1 Inhibitors[Title/Abstract])) OR (PD-1 Inhibitor[Title/Abstract])) OR (Inhibitor, PD-1[Title/Abstract])) OR (PD 1 Inhibitor[Title/Abstract])) OR (Programmed Cell Death Protein 1 Inhibitor[Title/Abstract])) OR (Programmed Cell Death Protein 1 Inhibitors[Title/Abstract])) OR (Surplulimab[Title/Abstract])) OR (Pembrolizumab[Title/Abstract])) OR (Zimberelimab[Title/Abstract])) OR (Tislelizumab[Title/Abstract])) OR (Camrelizhumab[Title/Abstract])) OR (Sintilimab[Title/Abstract])) OR (Toripalimab[Title/Abstract])) OR (Nivolumab[Title/Abstract])) OR (Sugemalimab[Title/Abstract])) OR (Envafolimab[Title/Abstract])) OR (Atezolizumab[Title/Abstract])) OR (Durvalumab[Title/Abstract])) OR (Ipilimumab[Title/Abstract]))) (n=2394)

**Search strategy in cochrane library**

cochrane library search:

#1 MeSH descriptor: [Carcinoma, Hepatocellular] explode all trees (n=2417)

#2 (Carcinoma, Hepatocellular or Carcinomas, Hepatocellular or Hepatocellular Carcinomas or Liver Cell Carcinoma, Adult or Liver Cancer, Adult or Adult Liver Cancer or Adult Liver Cancers or Cancer, Adult Liver or Cancers, Adult Liver or Liver Cancers, Adult or Liver Cell Carcinoma or Carcinoma, Liver Cell or Carcinomas, Liver Cell or Cell Carcinoma, Liver or Cell Carcinomas, Liver or Liver Cell Carcinomas or Hepatocellular Carcinoma or Hepatoma or Hepatomas):ti,ab,kw (n=11758)

#3 #1 or #2 (n=11758)

#4 MeSH descriptor: [Immune Checkpoint Inhibitors] explode all trees (n=199)

#5 (Immune Checkpoint Inhibitors or Checkpoint Inhibitors, Immune or Immune Checkpoint Inhibitor or Checkpoint Inhibitor, Immune or Immune Checkpoint Blockers or Checkpoint Blockers, Immune or Immune Checkpoint Blockade or Checkpoint Blockade, Immune or Immune Checkpoint Inhibition or Checkpoint Inhibition, Immune or PD-L1 Inhibitors or PD L1 Inhibitors or PD-L1 Inhibitor or PD L1 Inhibitor or Programmed Death-Ligand 1 Inhibitors or Programmed Death Ligand 1 Inhibitors or PD 1 PD L1 Blockade or CTLA-4 Inhibitors or CTLA 4 Inhibitors or CTLA-4 Inhibitor or CTLA 4 Inhibitor or Cytotoxic T-Lymphocyte-Associated Protein 4 Inhibitors or Cytotoxic T Lymphocyte Associated Protein 4 Inhibitors or Cytotoxic T-Lymphocyte-Associated Protein 4 Inhibitor or Cytotoxic T Lymphocyte Associated Protein 4 Inhibitor or PD-1 Inhibitors or PD 1 Inhibitors or PD-1 Inhibitor or Inhibitor, PD-1 or PD 1 Inhibitor or Programmed Cell Death Protein 1 Inhibitor or Programmed Cell Death Protein 1 Inhibitors or Surplulimab or Pembrolizumab or Zimberelimab or Tislelizumab or Camrelizhumab or Sintilimab or Toripalimab or Nivolumab or Sugemalimab or Envafolimab or Atezolizumab or Durvalumab or Ipilimumab):ti,ab,kw (n=13730)

#6 #4 or #5 (n=13730)

#7 #3 and #6 (n=934)

**Search strategy in Web of Science**

Web of Science search:

(TS=(Carcinoma, Hepatocellular) OR AB=(Carcinoma, Hepatocellular OR Carcinomas, Hepatocellular OR Hepatocellular Carcinomas OR Liver Cell Carcinoma, Adult OR Liver Cancer, Adult OR Adult Liver Cancer OR Adult Liver Cancers OR Cancer, Adult Liver OR Cancers, Adult Liver OR Liver Cancers, Adult OR Liver Cell Carcinoma OR Carcinoma, Liver Cell OR Carcinomas, Liver Cell OR Cell Carcinoma, Liver OR Cell Carcinomas, Liver OR Liver Cell Carcinomas OR Hepatocellular Carcinoma OR Hepatoma OR Hepatomas)) AND (TS=(Immune Checkpoint Inhibitors) OR AB=(Immune Checkpoint Inhibitors OR Checkpoint Inhibitors, Immune OR Immune Checkpoint Inhibitor OR Checkpoint Inhibitor, Immune OR Immune Checkpoint Blockers OR Checkpoint Blockers, Immune OR Immune Checkpoint Blockade OR Checkpoint Blockade, Immune OR Immune Checkpoint Inhibition OR Checkpoint Inhibition, Immune OR PD-L1 Inhibitors OR PD L1 Inhibitors OR PD-L1 Inhibitor OR PD L1 Inhibitor OR Programmed Death-Ligand 1 Inhibitors OR Programmed Death Ligand 1 Inhibitors OR PD 1 PD L1 Blockade OR CTLA-4 Inhibitors OR CTLA 4 Inhibitors OR CTLA-4 Inhibitor OR CTLA 4 Inhibitor OR Cytotoxic T-Lymphocyte-Associated Protein 4 Inhibitors OR Cytotoxic T Lymphocyte Associated Protein 4 Inhibitors OR Cytotoxic T-Lymphocyte-Associated Protein 4 Inhibitor OR Cytotoxic T Lymphocyte Associated Protein 4 Inhibitor OR PD-1 Inhibitors OR PD 1 Inhibitors OR PD-1 Inhibitor OR Inhibitor, PD-1 OR PD 1 Inhibitor OR Programmed Cell Death Protein 1 Inhibitor OR Programmed Cell Death Protein 1 Inhibitors OR Surplulimab OR Pembrolizumab OR Zimberelimab OR Tislelizumab OR Camrelizhumab OR Sintilimab OR Toripalimab OR Nivolumab OR Sugemalimab OR Envafolimab OR Atezolizumab OR Durvalumab OR Ipilimumab))。（n=3015）

**Search strategy in Embase**

**Embase search:**

#1 'carcinoma, hepatocellular':ab,ti OR 'carcinomas, hepatocellular':ab,ti OR 'hepatocellular carcinomas':ab,ti OR 'liver cell carcinoma, adult':ab,ti OR 'liver cancer, adult':ab,ti OR 'adult liver cancer':ab,ti OR 'adult liver cancers':ab,ti OR 'cancer, adult liver':ab,ti OR 'cancers, adult liver':ab,ti OR 'liver cancers, adult':ab,ti OR 'liver cell carcinoma':ab,ti OR 'carcinoma, liver cell':ab,ti OR 'carcinomas, liver cell':ab,ti OR 'cell carcinoma, liver':ab,ti OR 'cell carcinomas, liver':ab,ti OR 'liver cell carcinomas':ab,ti OR 'hepatocellular carcinoma':ab,ti OR hepatoma:ab,ti OR hepatomas:ab,ti （n=201312）

#2 'immune checkpoint inhibitors':ab,ti OR 'checkpoint inhibitors, immune':ab,ti OR 'immune checkpoint inhibitor':ab,ti OR 'checkpoint inhibitor, immune':ab,ti OR 'immune checkpoint blockers':ab,ti OR 'checkpoint blockers, immune':ab,ti OR 'immune checkpoint blockade':ab,ti OR 'checkpoint blockade, immune':ab,ti OR 'immune checkpoint inhibition':ab,ti OR 'checkpoint inhibition, immune':ab,ti OR 'pd-l1 inhibitors':ab,ti OR 'pd l1 inhibitors':ab,ti OR 'pd-l1 inhibitor':ab,ti OR 'pd l1 inhibitor':ab,ti OR 'programmed death-ligand 1 inhibitors':ab,ti OR 'programmed death ligand 1 inhibitors':ab,ti OR 'pd 1 pd l1 blockade':ab,ti OR 'ctla-4 inhibitors':ab,ti OR 'ctla 4 inhibitors':ab,ti OR 'ctla-4 inhibitor':ab,ti OR 'ctla 4 inhibitor':ab,ti OR 'cytotoxic t-lymphocyte-associated protein 4 inhibitors':ab,ti OR 'cytotoxic t lymphocyte associated protein 4 inhibitors':ab,ti OR 'cytotoxic t-lymphocyte-associated protein 4 inhibitor':ab,ti OR 'cytotoxic t lymphocyte associated protein 4 inhibitor':ab,ti OR 'pd-1 inhibitors':ab,ti OR 'pd 1 inhibitors':ab,ti OR 'pd-1 inhibitor':ab,ti OR 'inhibitor, pd-1':ab,ti OR 'pd 1 inhibitor':ab,ti OR 'programmed cell death protein 1 inhibitor':ab,ti OR 'programmed cell death protein 1 inhibitors':ab,ti OR surplulimab:ab,ti OR pembrolizumab:ab,ti OR zimberelimab:ab,ti OR tislelizumab:ab,ti OR camrelizhumab:ab,ti OR sintilimab:ab,ti OR toripalimab:ab,ti OR nivolumab:ab,ti OR sugemalimab:ab,ti OR envafolimab:ab,ti OR atezolizumab:ab,ti OR durvalumab:ab,ti OR ipilimumab:ab,ti（n=80553）

#3 #1 AND #2（n=3590）
