# Supplementary figures and images for: Hepatotoxicity of ICI monotherapy or combination therapy in HCC: A systematic review and meta-analysis
Source: PLoS One. 2025 May 29;20(5):e0323023. doi: 10.1371/journal.pone.0323023 (PMC12121757; doi:10.1371/journal.pone.0323023)

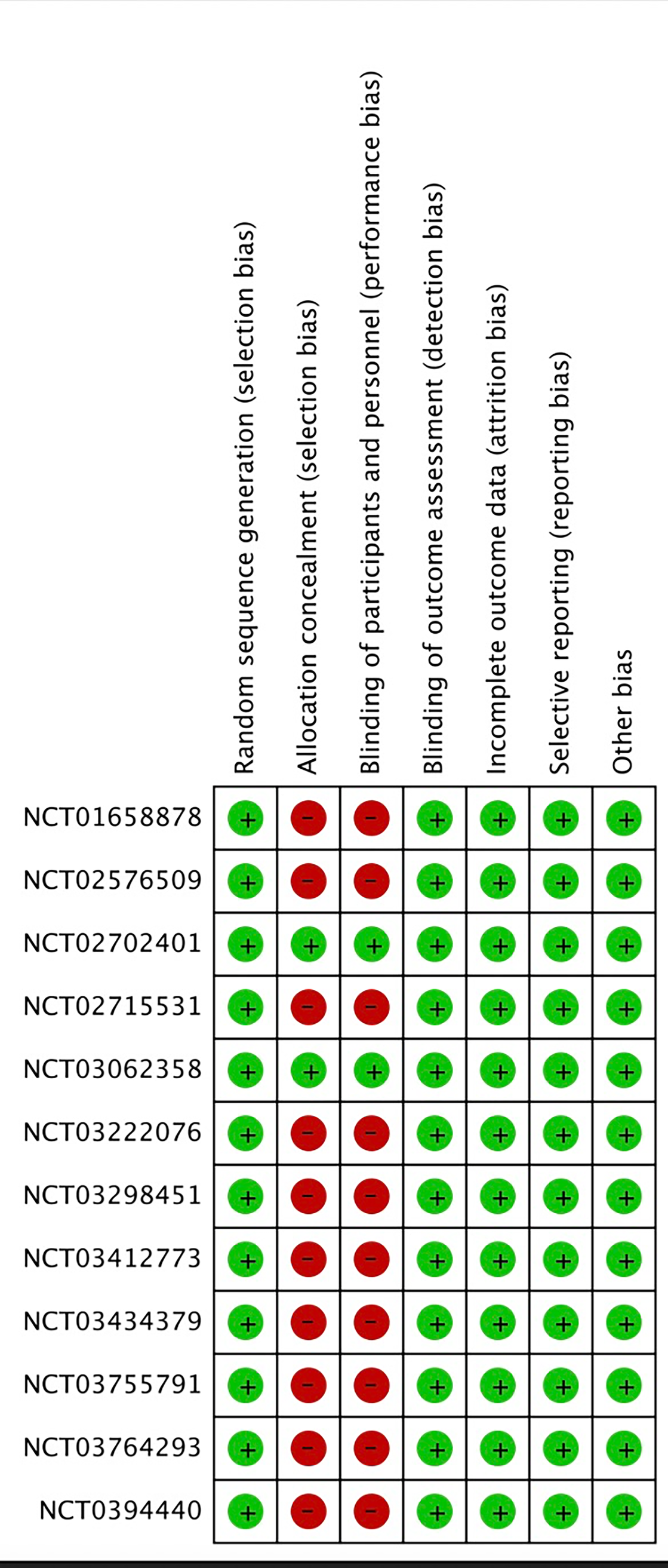

Supplement: S2 Fig — (TIF) [file pone.0323023.s002.tif]

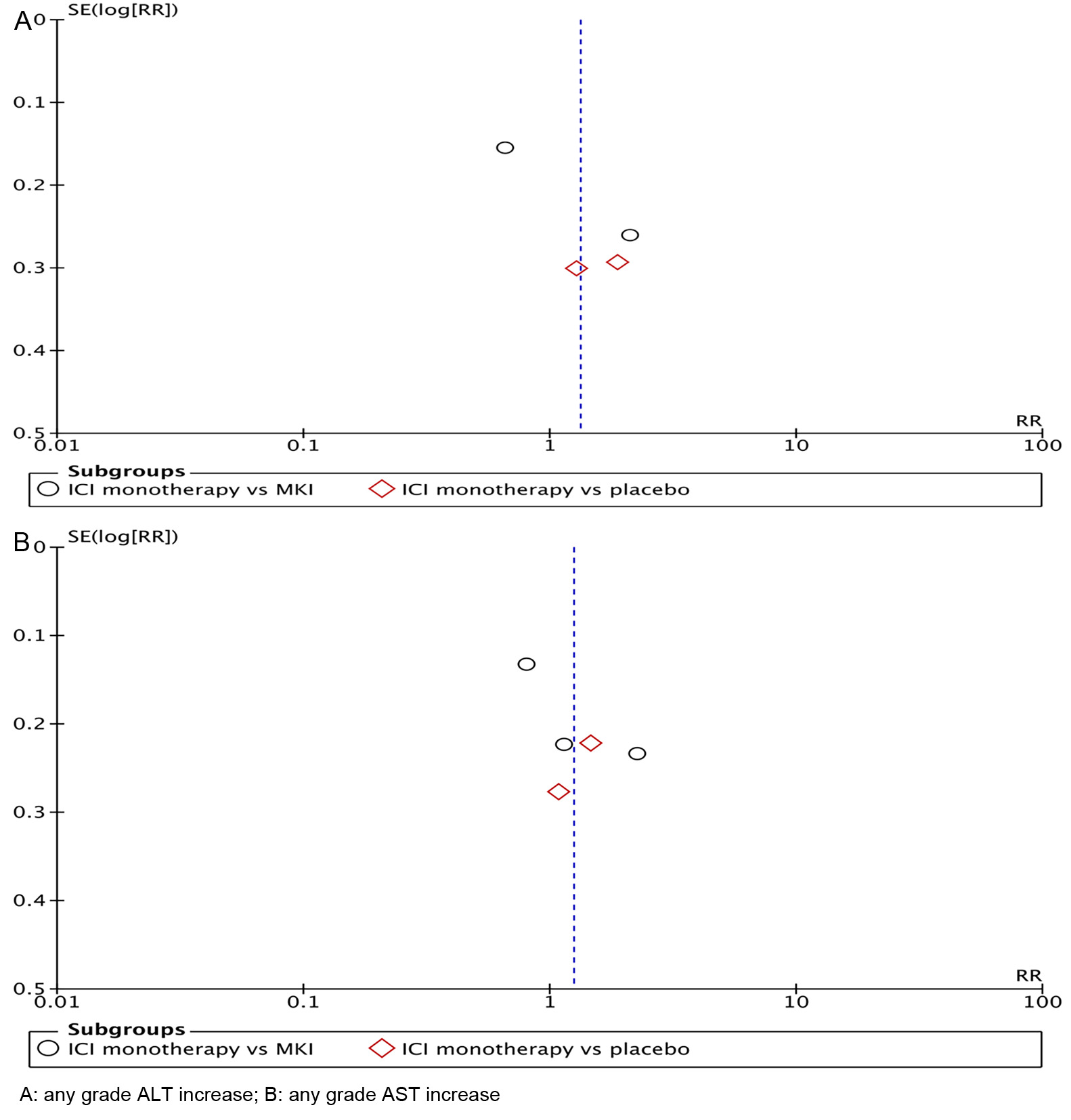

Supplement: S3 Fig — (TIF) [file pone.0323023.s003.tif]

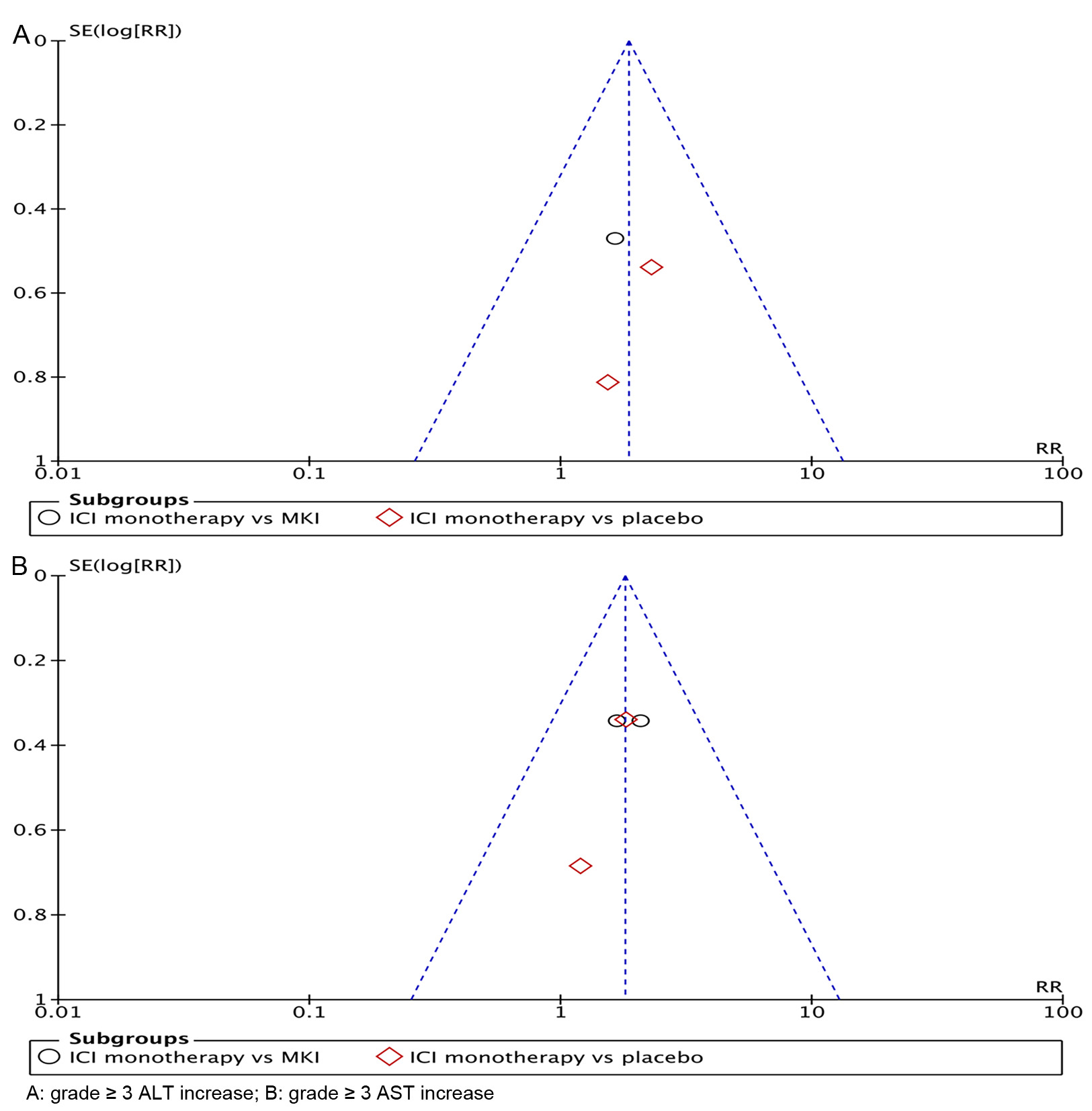

Supplement: S4 Fig — (TIF) [file pone.0323023.s004.tif]

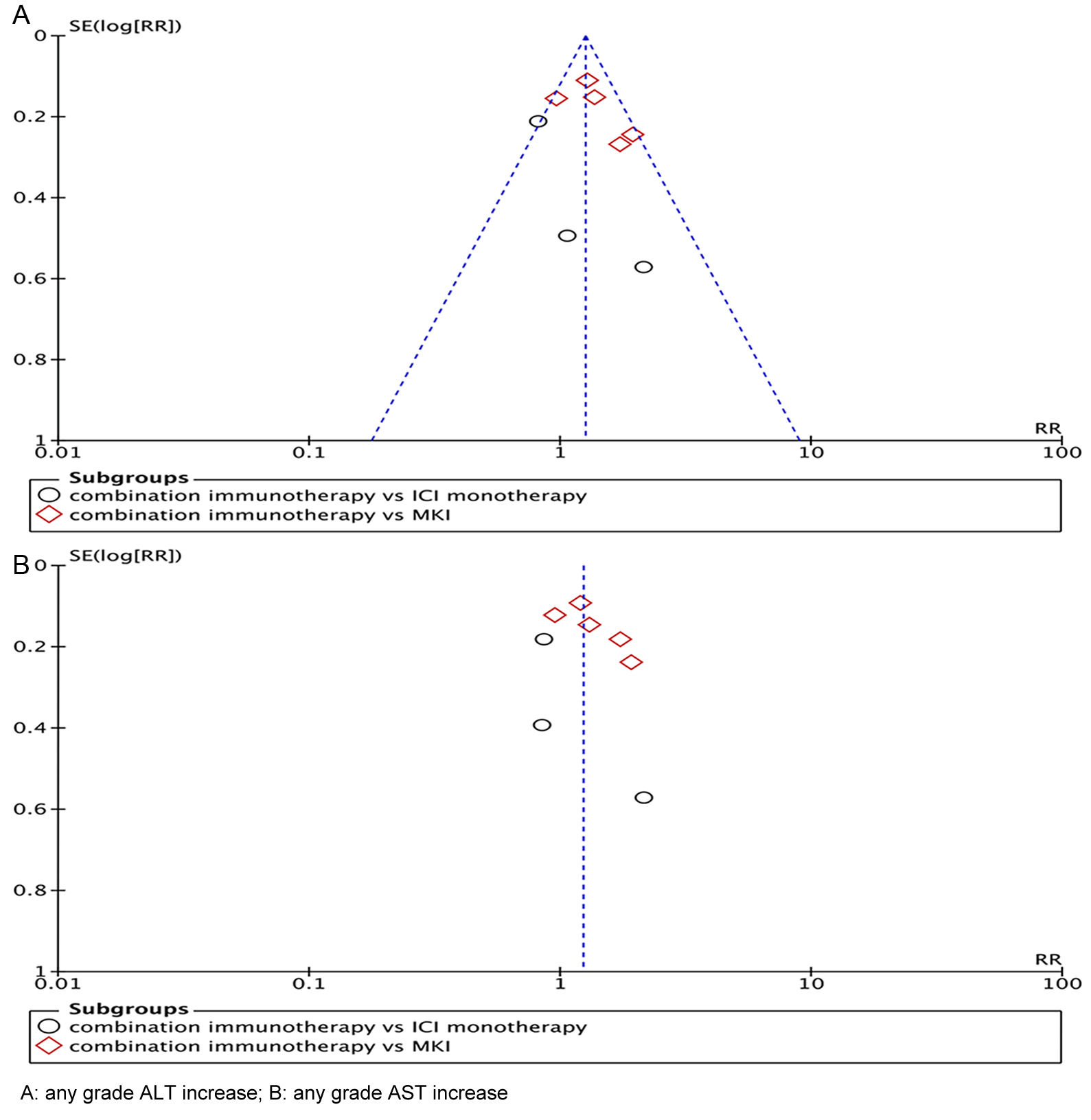

Supplement: S5 Fig — (TIF) [file pone.0323023.s005.tif]

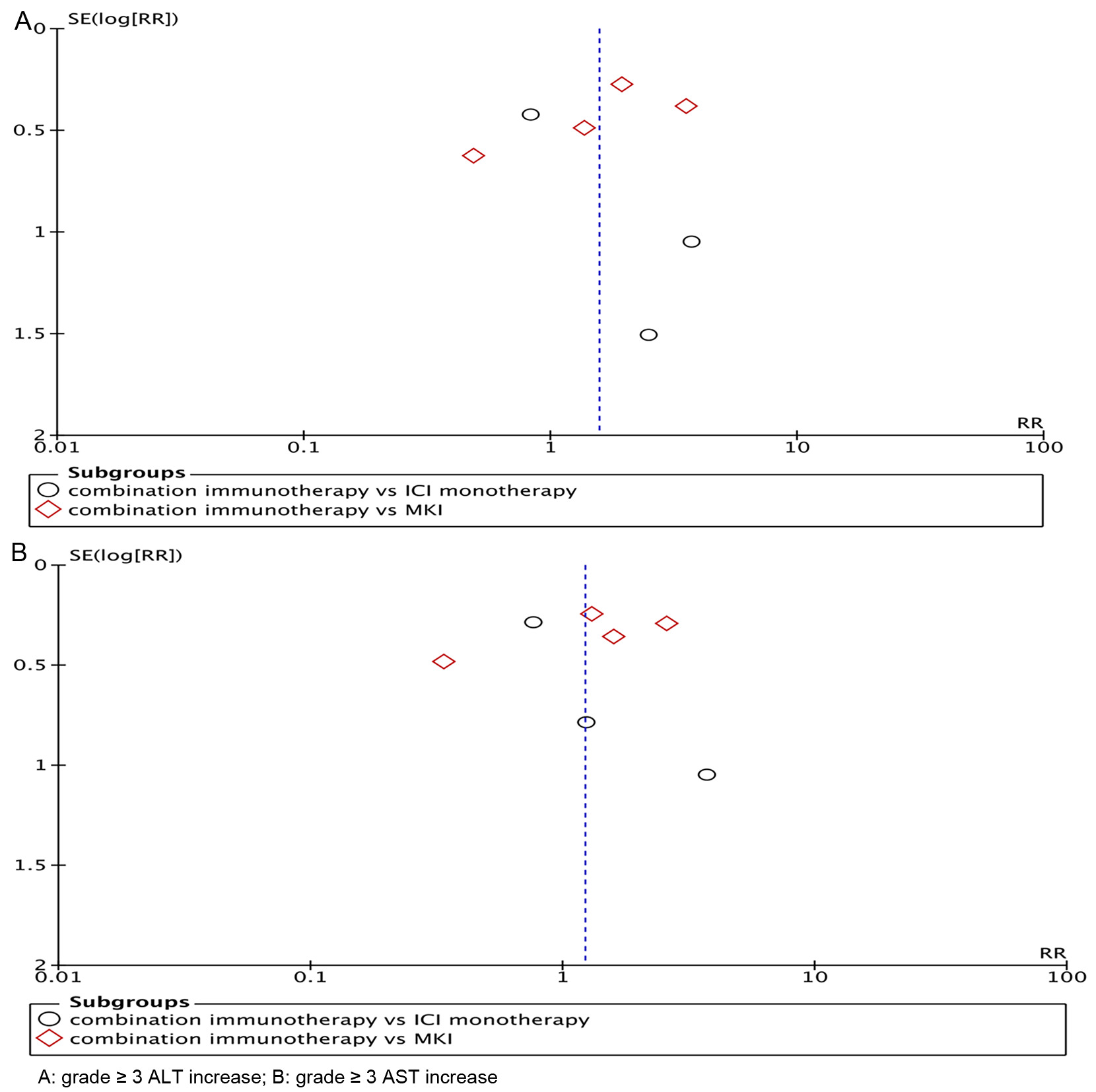

Supplement: S6 Fig — (TIF) [file pone.0323023.s006.tif]

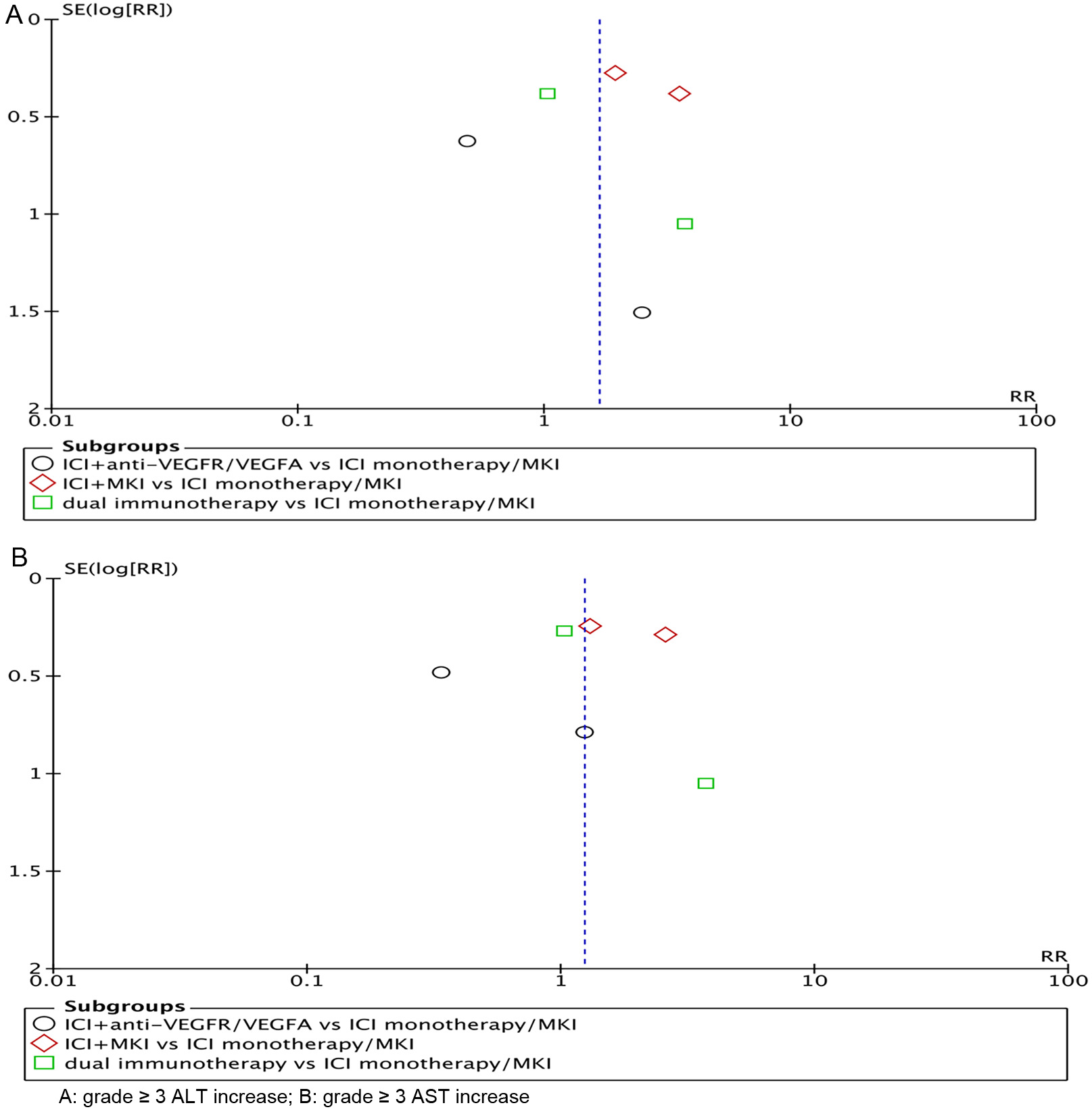

Supplement: S7 Fig — (TIF) [file pone.0323023.s007.tif]
